# Clinical Presentation as a Predictor of the Response to Methotrexate Therapy in Patients with Ectopic Pregnancy

**DOI:** 10.1155/2022/5778321

**Published:** 2022-11-28

**Authors:** Sarah Almutairy, Lateefa Othman Aldakhil

**Affiliations:** ^1^College of Medicine, King Saud University, Riyadh, Saudi Arabia; ^2^OB/Gyn Department King Khalid Hospital, College of Medicine, King Saud University, Riyadh, Saudi Arabia

## Abstract

**Purpose:**

Ectopic pregnancy can be fatal if not diagnosed and timely treated. There is an increase in ectopic pregnancy rate which attributes in part to fertility medications and procedures and early diagnosis. Methotrexate, a folic acid antagonist, is widely used in the medical treatment of ectopic pregnancy. Many studies examined the safety and success rate of methotrexate looking into factors affecting the success rate, if the patient may present with symptoms such as abdominal pain, and some consider this as impeding rupture and it might affect the success of medical treatment. This study evaluates the success rate of methotrexate treatment outcomes in regard to presentation and looks into other factors that can help choosing a single or multiple dose modality.

**Methods:**

This is a retrospective review of 154 cases of ectopic pregnancy treated with methotrexate from January 2011 to December 2020 at King Khalid University Hospital (KKUH). Demographic data, clinical presentation, treatment progress, and outcome and failure rate were collected and analyzed. Student's *t*-test was used for statistical analysis of associations in SPSS.

**Results:**

154 patients were treated with MTX; of those patients, 25 received more than one dose. The difference between the responses to MTX treatment in symptomatic and asymptomatic individuals was not significant (*p* = 0.267). 131 (85%) had successful treatment. There were no associations between patient BMI, ectopic mass size, or ectopic mass site, the presence or absence of pelvic fluid on ultrasound at diagnosis, and the treatment success rate. There was a significant decline in the treatment success rate with increasing *β*-hCG levels on the presentation day (*p* = 0.035) and on day 4 (*p* value <0.001) of treatment.

**Conclusion:**

MTX treatment can be used to manage symptomatic patients with ectopic pregnancy. The success rate in symptomatic patients is not different from that in asymptomatic patients. *β* − hCG levels > 5000 IU/L. Pretreatment and on day 4 posttreatment is associated with higher failure rate.

## 1. Introduction

Ectopic pregnancy is an acute emergency in obstetrics and gynecology that can be fatal if not diagnosed and treated in a timely manner. The reported incidence of ectopic pregnancy is approximately 2%, and it is a leading cause of maternal death, accounting for 10% of all maternal deaths [1]. The incidence has increased over the last few years due to an uptick in assisted reproductive technology use and the prevalence of pelvic inflammatory diseases [2, 3]. Advances in ultrasound technology and rapid serum hormone assays with high sensitivity have enabled early diagnosis before the presentation of symptoms, thus reducing the risk of tubal rupture and increasing the probability of success with more conservative medical treatments [4, 5].

In the Kingdom of Saudi Arabia (KSA), a 33-year retrospective study reported an incidence of 0.5% for ectopic pregnancies [6]. Previous abortion, pelvic surgery, previous ectopic pregnancy, infertility treatments, and uterine fibroids have been identified as important risk factors for ectopic pregnancy among Saudi females [7].

Timely diagnosis and appropriate treatment can reduce the maternal morbidity and mortality associated with ectopic pregnancy. Traditional salpingectomy is a reliable treatment approach for ectopic pregnancy; however, the loss of fallopian tubes and the inherent risk of surgical intervention make this therapeutic approach less desirable for gynecologists around the globe. Therapeutic management can reduce tubal damage and prevent the inherent risks of surgical interventions. Recently, technological advancements have allowed the early detection of ectopic pregnancies and their subsequent treatment [8].

MTX is a folic acid antagonist that inhibits cell proliferation. MTX prevents cytotrophoblast proliferation in ectopic gestation, leading to the reduced production of beta-human chorionic gonadotropin (*β*-hCG). The levels of progesterone secreted by the corpus luteum are thus decreased [9]. MTX has been proven to be very effective for treating ectopic pregnancies [10]. The protocols for the management of ectopic pregnancies with MTX include single-dose, 2-dose, and multiple-dose protocols [11, 12]. MTX has been shown to be effective for the treatment of ectopic pregnancy. However, treatment is recommended only for hemodynamically stable patients who present with an adnexal mass and initial *β*-hCG level in a specific range. However, there is an ongoing debate regarding the management of hemodynamically stable patients with MTX who do not meet the aforementioned criteria but want to avoid surgery. *β*-hCG levels are also used as prognostic markers after MTX treatment. The success rate of MTX treatment is variable, ranging from 63% to 97%, depending on the treatment protocol and patient response [13]. MTX treatment has been used widely for ectopic pregnancies in KSA, and the success rate of this medical treatment was shown to be 71% among Saudi females [14].

This study is aimed at investigating the success rate of MTX treatment in King Khalid University Hospital (KKUH) and identifying the effect of patients clinical presentation and other factors that can predict and influence therapeutic success of medical treatment in Saudi patients.

## 2. Methods

### 2.1. Study Design

This was a retrospective cohort study conducted at a major tertiary care center in Riyadh after approval was obtained from the research ethics committee. The study included 154 reported cases of ectopic pregnancy over ten years from January 2011 to December 2020. All patients received MTX, had tubal ectopic pregnancies, and received medical management. The subjects were included in the study after receiving complete patient counseling regarding the risks and benefits of each management modality. Full explanations of the management plan and follow-up plan were provided to the patients, and written consent was obtained from the patients.

### 2.2. Inclusion Criteria

The study included all subjects diagnosed with tubal ectopic pregnancies who were managed with MTX. Patients were included after the diagnosis of an ectopic pregnancy based on the combination of two clinical signs and symptoms, including abdominal pain, vaginal bleeding, and ultrasonic findings suggestive of a tubal ectopic pregnancy; these ultrasonic findings included either a viable or nonviable adnexal mass, the presence of fluid in the pouch of Douglas, or an increasing B-hCG level with a pattern suggestive of an ectopic pregnancy.

### 2.3. Exclusion Criteria

Subjects with nontubal ectopic pregnancies and patients who received either conservative or initial surgical management were excluded from the study. Patients presenting with contraindications to MTX treatment, including hemodynamically unstable patients, patients with hematological, renal, hepatic, or pulmonary diseases, and patients with active peptic ulcers, were excluded. Patients with hypersensitivity to MTX, those who were breastfeeding, those who were unable to continue outpatient follow-up after receiving MTX, and those without access to the emergency room were also excluded.

### 2.4. Data Retrieval

Prior to 2015, hospital management collected patient records in the form of hard copies; for patients who presented before May 2015, the data were retrieved by reviewing medical charts. For patients presenting after May 2015, the data were collected from computerized medical records.

#### 2.4.1. Laboratory Analysis

After admission, laboratory investigations were carried out for complete blood count, liver enzymes, serum creatinine level quantification, and blood group status.

#### 2.4.2. Medical Treatment Protocol

Patients received either a single-dose protocol of 50 mg/m^2^ intramuscular MTX or a two-dose protocol of intramuscular 50 mg/m^2^ MTX on days 0 and 4 additional doses of methotrexate were given on day 7 and/or day 11 if hCG levels did not decrease by 15% during the follow-up period. Patients with viable tubal ectopic pregnancies received potassium chloride in addition to MTX therapy. Single-dose protocol or 2-dose protocol of methotrexate (day 0 and day 4) was based on treating physician preference. Patients in the Rh-negative blood group received anti-D after MTX administration. Failed medical treatment was considered if *β*-hCG did not decrease by 15% or more at follow-up period. The majority of patients were discharged on day 1 after receiving MTX management.

The patients' *β*-hCG levels were measured on days 4 and 7, followed by weekly analysis during outpatient clinics until these levels dropped to 0. Between days 4 and 7, patients with a ruptured ectopic pregnancy and plateauing or increasing *β*-HCG levels at any time of treatment duration were managed with surgical intervention. Successful MTX treatment was defined as complete resolution of an ectopic pregnancy and amelioration of the need for surgical intervention.

#### 2.4.3. Statistical Analysis

After quality checks on the dataset were performed, descriptive statistics were calculated. Measures for continuous variables are presented as the mean and standard deviation for normally distributed data, and categorical variables are presented as numbers and percentages. We used the chi-square test or Fisher's exact test to assess the significance of the associations between categorical variables. Correlations between various continuous variables were calculated using Spearman's correlation coefficient. A *p* value of less than 0.05 was considered statistically significant. All data analysis was performed using SPSS version v.23 (IBM Corp, Armonk, NY, USA).

## 3. Results

### 3.1. Baseline Data

One hundred fifty-five ectopic pregnancies were diagnosed during the ten-year period, at an incidence of 1.3%. One patient is unstable and require immediate surgery. The majority of women were 21–35 years old (76%), with an overall mean age of 31 years (std deviation 5.8, range “*r*”: 19–65; see Table 1). The mean gestational age was 6.4 weeks. Previous abortion (65 patients; 41.9%) and pelvic surgery (22 patients; 14.3%) were the most common risk factors. Lower abdominal pain and vaginal bleeding were present in 81 (52.6%) and 47 (30.5%) patients, respectively, and 98 (63.6%) patients in our cohort were symptomatic.

### 3.2. Response to Methotrexate Therapy

One hundred fifty-four patients were managed medically. 129 (84%) patients received single-dose MTX and 25 (16%) received two or more doses. Overall success rate was 85% with 106 (82%) in the single-dose regimen, while 23 (15.9%) patients required surgery for failed medical management, the success rate was 100% in the 25 patients who received 2-dose regimen of MT. Only two (1.3%) patients received transfusion, and no deaths were noted.

One patient had a viable ectopic and local injection of KCL potassium chloride long with systemic methotrexate. The difference between the response to MTX treatment in patients presenting with abdominal pain versus asymptomatic individuals was not significant (*p* = 0.267), as shown in Table 2. Similarly, BMI was also not shown to be associated with treatment success (p = 0.229), as shown in Table 1.

No significant correlation was found between treatment success and various ultrasound findings, including the presence of pelvic fluid (*p* = 0.95), ectopic mass site (*p* = 0.93), or ectopic mass size in cm (*p* = 0.708), as shown in Table 3.

### 3.3. Relationship between *β*-HCG Changes and Treatment Outcomes

We observed a significant decline in the treatment success rate with increasing *β*-HCG levels on the day of presentation (*p* = 0.035) and day 4 (*p* = <0.001), as shown in Table 4. There was no failure in the patients managed with the “2-dose” regimen.

## 4. Discussion

Ectopic pregnancy is a serious gynecological emergency. We observed an incidence of 1.3% for ectopic pregnancies during the ten-year study period from 2011 to 2020. No survey has reported the national-level incidence of ectopic pregnancies or associated demographics for KSA. However, various studies have reported data from separate tertiary care centers across different regions of the KSA. A previous study from Saudi Arabia estimated the incidence of ectopic pregnancies to be 1.19% during a ten-year study period from 2000 to 2011 [15]. This implies that the incidence of ectopic pregnancies has not increased significantly over the last few decades. The age range for previous studies was between 15 and 45 years [16, 17]. Most of the subjects in our study were aged from 21 to 35 years. This is close to the average age of 28.9 years, with most subjects in the age range of <25 years to 35 years. Previous abortion and pelvic surgery have been identified as significant risk factors for ectopic pregnancy in previously published studies [7].

MTX is an effective treatment for early unruptured ectopic pregnancy and is usually preferred over surgical interventions. MTX can be used to treat ectopic pregnancies that occur in cervical, cornual, and cesarean scar locations without any adverse effects on a patient's ovarian reserve or subsequent fertility [11]. The success rate of MTX treatment in our single-center study was 85.1%. Previous reports from different regions of the globe suggest similar success rates with MTX treatment that vary from 65–95%, with a mean rate of 82% in various populations [18–20]. These findings, combined with the results of previous studies, suggest that the success rates of MTX treatment for ectopic pregnancies are not ethnicity-dependent [14]. Furthermore, our data showed that patients' presentation did not correlate with the result of medical treatment. Several MTX treatment regimens are available; MTX can be administered as a single systemic dose regimen, 2-dose, or a multiple-dose regimen. The single-dose treatment regimen has fewer side effects, reduced overall costs, and improved patient compliance [21, 22]. The efficacy of tow or multiple-dose regimen is reportedly higher than that of a single-dose regimen in terms of treatment success and time-to-success [22, 23]. Among our study subjects, the 2-dose treatment regimen has 100% success rate and the failure rate in our single regimen was significantly higher in patients with initial *β* − hCG levels > 5000 IU/L. which made the 2-dose regimen a better approach for such patients, this regimen is shown to be safe and effective for the medical management of ectopic pregnancy [12, 23]. No significant association was found between BMI nor age and the treatment response or success rate of MTX treatment. The results of previous studies are mixed. Obese women at an increased risk of ectopic pregnancy [24]. An inverse relationship has been reported for BMI and the risk of ectopic pregnancy following assisted reproductive techniques [25]. Obesity and increase age were identified as a risk factor for a second MTX dose or surgical interventions [26, 27]. Furthermore, we did not find any association between the size or site of the ectopic mass and the presence of fluid in the pelvis with the success of treatment for ectopic pregnancy, which contrasts with previous reports [17, 27].

*β*-hCG levels have been the key prognostic biomarker for predicting and assessing the success of MTX treatment [20, 27, 28]. There is no consensus on the threshold value that best predicts success or failure, however many data use 5000 IL/L as a threshold for higher failure rate [11].

The success rate for patients with initial *β*-hCG concentrations less than the cutoff value of 4000 IU/L was significantly higher than that of patients with initial *β*-hCG concentrations more elevated than this cutoff value [16]. ACOG consider high initial *β*-hCG l level a relative contraindication to medical treatment. Systematic review evidence shows a failure rate of 14.3% or higher with methotrexate when initial B-hCG levels are higher than 5,000 IU/L compared with a 3.7% failure rate for B-hCG levels less than 5,000 IU/L [29]. *β*-hCG levels were found to be strongly correlated to the depth of trophoblastic invasion in the tubal wall [30]. which explained the higher failure rate associated with high initial *β*-hCG. The reported OR for failure is 5.45 (95% CI, 3.04–9.78) when initial *β*-hCG values are above 5,000 IU/L compared with that observed when *β*-hCG concentrations are below that threshold [11]. Most of the studies often have excluded patients from methotrexate treatment when *β*-hCG levels are greater than 5,000 IU/L, which may affect the results [8]. Our data showed a similar finding of high failure rate if initial *β*-hCG level is higher than 5,000 IU/L. also it showed that high level at day 4 posttreatment is significant for increasing the failure rate. As recommended by previous data, 2-dose or multiple dose regimens should be considered [8, 11, 28].

## 5. Prospects

MTX is an effective therapeutic option for ectopic pregnancy. However, timely detection is a prerequisite for the success of MTX treatment. High-risk individuals, such as those with a history of abortion and pelvic surgery, should be continuously monitored for signs of ectopic pregnancy to ensure early diagnosis. Furthermore, the outcome of medical treatment is not affected by the patients presenting symptoms.

## 6. Conclusion

MTX treatment success rate was not affected by patient clinical presentation, the treatment efficacy in patients typically presenting with abdominal pain and asymptomatic patients is similar. In patients with initial *β*-hCG or day 4 *β*-hCG posttreatment levels >5000 IU/L failure rate is higher and 2-dose regimen seems a better option.

## Figures and Tables

**Table 1:** Demographic characteristics.

Methotrexate treatment success		*N*	Mean	Std. deviation	*p* value
Age	Yes	131	31.37	5.851	0.122
	No	23	29.39	5.442	

Gestational age	Yes	130	6.47	1.744	0.797
	No	23	6.57	1.619	

BMI	Yes	131	28.556	6.7585	0.130
	No	23	26.304	5.1384	

**Table 2:** Symptomatic vs. asymptomatic comparison with successful treatment using the chi-squared test.

Symptomatic	Successful treatment		Total
	No	Yes	
No	6 (10.7%)	50 (89.3%)	56 (36.4%)
Yes	17 (15.3%)	81 (82.7%)	98 (63.6%)
		*p* value	0.267

**Table 3:** Association of USS findings and treatment success.

Methotrexate treatment success	USS findings							
	Fluid in the pelvis		Mass site		Adnexal mass size			
	No	Yes	Left	Right	<3 m	3-4 cm	5-6	>6
No	17 (15)	6 (14.6)	12 (15)	11 (15.5)	8 (19)	12 (14.8)	2 (8.3)	1 (16.7)
Yes	96 (85)	35 (85.4)	68 (85)	60 (84.5)	34 (81)	69 (85.2)	22 (91.7)	5 (83.3)
Total	113 (73.4)	41 (26.6)	80 (53)	71 (47)	41 (27.5)	81 (52.9)	24 (15.7)	6 (3.9)
*p* value	**0.95**		0.93		**0.708**			

**Table 4:** Association of *β*-hCG values on days 0 and 4 in relation to methotrexate treatment success.

Methotrexate treatment success	*β*-hCG level at day zero			*β*-hCG level at day 4		
	No	Yes	Total	No	Yes	Total
<5000	18 (12.9)	121 (87.1)	139 (90.3)	10 (7.8)	118 (92.2)	128 (83.1)
≥5000	5 (33.3)	10 (66.7)	15(9.7)	13 (50.0)	13 (50.0)	26 (16.9)
*p* value	0.035			<0.0001		

## Data Availability

Data are available from the authors upon request and with permission of King Khalid University Hospital.
